# Exosomes and autophagy in ocular surface and retinal diseases: new insights into pathophysiology and treatment

**DOI:** 10.1186/s13287-022-02854-8

**Published:** 2022-05-03

**Authors:** Shisi Ma, Xiao Liu, Jiayang Yin, Lili Hao, Yuyao Diao, Jingxiang Zhong

**Affiliations:** 1 Department of Ophthalmology, The First Affiliated Hospital of Jinan University, Jinan University, 613 West Huangpu Ave, Guangzhou, 510632 Guangdong China; 2 The Sixth Affiliated Hospital of Jinan University, Jinan University, Dongguan, Guangdong China

**Keywords:** Autophagy, Exosomes, Extracellular vesicles, Ocular surface diseases, Ocular pathophysiology, Retinal diseases

## Abstract

**Background:**

Ocular surface and retinal diseases are widespread problems that cannot be ignored in today’s society. However, existing prevention and treatment still have many shortcomings and limitations, and fail to effectively hinder the occurrence and development of them.

**Main body:**

The purpose of this review is to give a detailed description of the potential mechanism of exosomes and autophagy. The eukaryotic endomembrane system refers to a range of membrane-bound organelles in the cytoplasm that are interconnected structurally and functionally, which regionalize and functionalize the cytoplasm to meet the needs of cells under different conditions. Exosomal biogenesis and autophagy are two important components of this system and are connected by lysosomal pathways. Exosomes are extracellular vesicles that contain multiple signaling molecules produced by multivesicular bodies derived from endosomes. Autophagy includes lysosome-dependent degradation and recycling pathways of cells or organelles. Recent studies have revealed that there is a common molecular mechanism between exosomes and autophagy, which have been, respectively, confirmed to involve in ocular surface and retinal diseases.

**Conclusion:**

The relationship between exosomes and autophagy and is mostly focused on fundus diseases, while a deeper understanding of them will provide new directions for the pathological mechanism, diagnosis, and treatment of ocular surface and retinal diseases.

## Background

Ocular surface diseases (OSDs) such as dry eye disease (DED), keratitis, and conjunctivitis, damaging the normal structure and function of the tear film, cornea, and conjunctiva, are very common eye disorders, and major causes of visual impairment [1]. With the increasing aging population and a growing incidence of diabetes worldwide, the treatment demand of retinal diseases including age-related macular degeneration (AMD), diabetic retinopathy (DR) are on the rise [2]. The high incidences of those diseases influence the life quality of the patient severely. However, existing prevention and treatment still have many shortcomings and limitations, and fail to effectively hinder the occurrence and development of OSDs and retinal diseases. It is crucial to explore a better alternative therapy, which scientists have pursued in recent years [3, 4]. The endomembrane system (EMS) and the membrane-trafficking system in eukaryotic cells containing the endoplasmic reticulum (ER), Golgi apparatus, lysosome, plasma membrane, endosome, and secretory vesicles are groups of related membrane-bound organelles [5]. EMS participates in the basic process of cellular activities, for instance, endocytosis, exocytosis, and signal transduction pathways. These lipid membranes are intertwined in structure and function. They are in various forms, fused or separated from each other, and their components are constantly updated [6]. Understanding the complex connections among them will improve our perception of intracellular vesicle transport, intracellular and intercellular communication in membrane compartments, and help us understand the basic intracellular mechanisms of OSDs and retinal diseases.

### Overview of exosomes

The International Society for Extracellular Vesicles indicates exosomes are 30–150 nm extracellular vesicles (EVs) derived from the endocytic pathway, which are also known for being involved in cell–cell communication by regulating physiological functions and/or pathological conditions through carrying lipids, proteins, or RNA as pieces of information toward cells in different distances [7]. Endocytosis (also known as transcytosis or endocytosis) is the process of transporting substances through the deformation and movement of the plasma membrane. It can be divided into phagocytosis, and receptor-mediated endocytosis based on different substances and mechanisms. These membranes invaginated and divided into intracellular vesicles. After fusion with the early endosomes, the primary endocytic vesicles begin to classify transported substances. Then the early endosomes experience series of biochemical transformations and mature to the late endosomes, finally fused with lysosomes [8]. During the maturation process, another membrane invagination and fission of some endosomes produce the intermediate organelles called multivesicular bodies (MVB), which contain many intraluminal vesicles (ILVs). Then, MVB directly fusing with the plasma membrane and transporting ILVs to the extracellular space can produce exosomes. Finally, exosomes can be internalized by secretory or other cells in paracrine and/or endocrine signaling pathways [9].

For the past few years, a growing amount of research points out that exosomes take part in intercellular communication and relate to physiological and pathological mechanisms in various conditions, involving immune regulation, tissue regeneration, viral infection, tumor occurrence, development and metastasis [10–12]. At the same time, research on the production and application of exosomes in disease diagnosis and target administration is also undergoing in-depth studies [13]. Therapies involving mesenchymal stem cells (MSCs), which are obtained from adipose, bone marrow, or umbilical cord tissues, have been shown to promote tissue repair [14–18]. With the advantages of immune compatibility, mass production and efficient transport capacity, exosomes derived from MSCs (MSC-Exos) have attracted the attention in academic circles, and has been used in the treatment of regenerative medicine and ocular diseases. The current treatment developments of ocular diseases focus on exosomes, cell transplantation, biopolymers, and other approaches [19, 20]. Exosomes not only participate in the occurrence and development of DED, corneal injury, corneal dystrophy, AMD, DR and retinal ischemia, but also have potential value in diagnosing and targeted treating them.

### Overview of autophagy

Autophagy, maintaining the balance among the synthesis, degradation and circulation of cellular contents, is a self-metabolic process in all eukaryotic cell lives [21]. It processes invading pathogens, denatured proteins and damaged organelles through the lysosomal degradation pathway. There is a low level of autophagy under normal physiological conditions, which is beneficial to substance circulation, energy exchanges and information transfer. While under certain circumstances such as starvation, hypoxia or inflammation, cause the imbalance of autophagy, disrupt the body's equilibrium states and lead to the occurrence of various diseases [22]. Macroautophagy, small autophagy and molecular chaperone-mediated autophagy constitute three forms of autophagy. The main difference of them lies in the way the cell contents are transported to the lysosome. Autophagy usually refers to macroautophagy, which is characterized by the transfer of phagocytosed intracellular proteins and organelles to lysosomes for digestion and degradation through autophagic vesicles with a double membrane structure [23]. The autophagy process is inseparable from the endolysosomal pathway. On the one hand, autophagosomes form intermediate vesicles (amphisomes) through fusing with late endosomes, which are eventually transformed into autolysosomes; on the other hand, autophagosomes form to autophagolysosomes directly by fusing with lysosomes. No matter which route is taken, autophagy lysosomes finally digest and decompose organelles and proteins through acid hydrolase [24, 25].

Interestingly, from the cornea to the retina, almost all ocular cells express autophagy-related proteins to varying degrees and rely on autophagy to maintain the normal structure and physiological functions [26]. Therefore, it is of great significance to maintaining the stability of the intracellular environment by adjusting the basic autophagy and regulating the level of autophagy during the ocular diseases.

### The interaction of autophagy and exosomes biogenesis

**Fig. 1:**
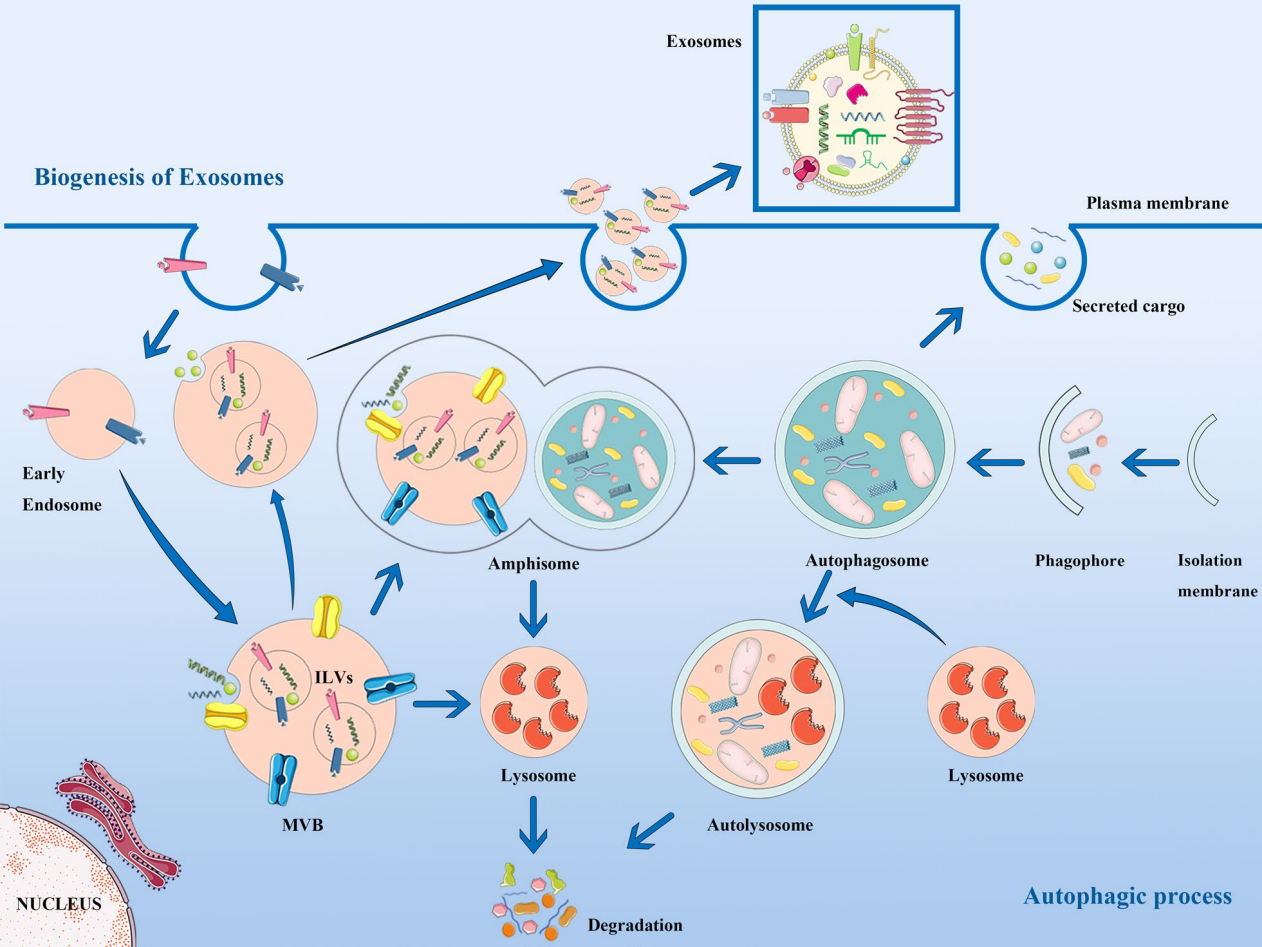
Crosstalk between the biogenesis of exosomes and the autophagic process. Representation of the emerging cooperation between exosomes biogenesis and autophagy helped by the amphisomes. Autophagosomes fuse with MVBs to form amphisomes which have two possible fates following the cell conditions. On the one hand, MVBs enrich in intraluminal vesicles (ILVs) can either fuse with autophagosome to form amphisomes inducing the autophagic degradation, or directly fuse with the plasma membrane to release EVs as exosomes out of the extracellular area, which contain abundant biological signaling materials like noncoding RNAs, DNAs and proteins. On the other hand, autophagy can also regulate the exosomes biogenesis through the intersection of amphisomes

Exosomal biogenesis and autophagy are two important components of EMS and share intricate molecular and regulatory mechanisms, indicating that these two processes are closely related and are of great significance to normal physiology and pathological states [27]. Exosomal-autophagy synergistic reaction maintains intracellular homeostasis by cellular secretions release and/or lysosome degradation. Based on existing research, the interaction between autophagy and EVs can be summarized in the following aspects: Firstly, autophagy and EVs share the common molecular mechanisms, including autophagy-related proteins and key proteins of EVs biogenesis family pathways; Secondly, the degradation of vesicles mediated by the binding of MVB and lysosomes is the result of EVs biogenesis and exosomes release the vesicle degradation; Third, the conventional way of autophagosomes is to be degraded into waste substrates, but when under different stimulating conditions, autophagosomes can carry related secreted cargos as a substitute for their degradation [28]. Figure 1 outlines the relationship between them.

Parts of the autophagy mechanism have been verified that are not helpful to the autophagic process itself but contribute to the biogenesis of exosomes. A recent study emphasized the particular key functions of the ATG5-ATG16L1 complex during exosomal biogenesis. ATG5 mediated the vacuolar proton pump (V1V0-ATPase) to dissociate from MVB, which promoted MVB to fuse with plasma membrane and the exosomes release. Therefore, knocked out ATG5 and/or ATG16L1 genes would not only reduce the release of exosomes but also weaken the exosomal enrichment to a large extent [29, 30]. Those studies have provided the speculation that the fate of MVB and following exosomal biogenesis are adjusted by autophagy-related proteins directly. The catalyst of LC3B, ATG12–ATG3 complex, which regulates the biogenesis of exosomes by affecting apoptosis-linked-gene-2 interacting protein X (ALIX), is also a protein related to the endosomal sorting complex required for transport (ESCRT). Here, the deficiency of ATG12–ATG3 complex changed the morphology of MVB, hindered the transportation of late endosomes and exosomes release. Furthermore, due to the knockdown of ALIX gene, the decreased basal level of autophagy flux demonstrated there was a cooperative adjustment between autophagy and exosomal biogenesis [31]. The mammalian Class III PI3K (PI3KC3) complex containing PIK3C3, Beclin1 and p150, which was bound up with various regulatory proteins to show different functions and necessary for autophagy and endocytosis [32]. When the PI3K complex was unstable, there would be decreased levels both in exosomal release and autophagic flux [33].

Therefore, EVs carry different cargos to induce autophagy of target cells by connected pathways, in the meantime, autophagy controls the EVs especially exosomes biogenesis and degradation [6]. The main advances about the respective therapeutic effects of autophagy, exosomes and the crosstalk between them in ocular diseases are summarized below.

## Research progress of exosomes and autophagy in ocular surface diseases

### Dry eye diseases

DED, accompanied by increased osmolarity and inflammation, is a common multifactorial ocular surface disease, resulting in tear film instability and visual disturbance [34]. In 2005, it was the first detection of Sjogren’s syndrome (SS)-specific autoantigens in exosomes indicated they might be involved in the presentation from intracellular autoantigens to autoreactive lymphocytes, which was a key step of SS development [35]. As a lacrimal inflammation related disease, the inflammation of lacrimal glands and the clinical evaluations were improved in SS rabbit models after subconjunctival injection of MSC-Exos [36]. Another study demonstrated that ophthalmitis caused by human SS was efficiently relieved by inhibiting the secretion of macrophage-related inflammatory cytokines interleukin-1 beta (IL-1β) and tumor necrosis factor-alpha (TNF-α), and increasing anti-inflammatory factors interleukin-10 (IL-10) and transforming growth factor-beta (TGF-β) after giving exosomes derived from human umbilical cord mesenchymal stem cells [37]. Therefore, MSC-Exos can be used for potential drug delivery vehicles in eye drops for SS-associated dry eyes [38]. MSC-Exos were also shown to be efficacious in treating graft-versus-host disease–associated dry eye disease via miR-204–mediated pathway [39]. Exosomes derived from mouse adipose-derived mesenchymal stem cells promote the reconstruction corneal epithelium, goblet cells, and tear secretion, reduce the levels of inflammatory cytokines IL-1β and interleukin-6 (IL-6) in the cornea and conjunctiva tissues of DED mouse models, suggesting that they could be used as a potential treatment [40].

Previous research demonstrated that the expression of autophagy marker transcription genes and proteins, autophagy-related protein 5 (ATG5) and light chain protein 3B (LC3B) were elevated in tears and conjunctival tissues of SS-DE patients, proposing that autophagy played a role in the pathogenesis of DED and autophagic markers could be used as new diagnostic and therapeutic biomarkers [41], especially the diagnostic ability of ATG5 in tear [42]. In the lacrimal glands of DED mice, electron microscopy observed that an increased number of autophagosomes, damaged mitochondria and the compressed endoplasmic reticulum. The increased autophagy marker proteins expression has verified that DED induced the expression of hypoxia inducible factor-1α, which put the lacrimal gland cells in a hypoxia stress condition. At the same time, autophagy was activated to prevent further damage to the acinar cells and maintained the normal function of the lacrimal gland [43]. Autophagy activation protected in vitro DED model of human corneal epithelial cells (HCECs) by preventing inflammatory response and promoting cell survival in a late reaction to hyperosmotic stress, which was enhanced by rapamycin [44]. Similarly, melatonin, a hormone with anti-inflammation and antioxidant features, triggers the expression of hemeoxygenase-1 to protect HCECs from oxidative damage and decrease inflammation in DED mice by maintaining the normal autophagy level [45]. Melatonin-loaded polymer polyvinyl caprolactam-polyvinyl acetate-polyethyleneglycol graft copolymer micelles ameliorated hyperosmolarity-induced ocular surface damage of DED via phosphated and tension homology deleted on chromosome ten-induced kinase 1-mediated mitophagy [46]. Trehalose, as an autophagy activator, also reduced the expression of pro-inflammatory mediators TNF-α, IL-1β, IL-6, and IL-8 via transcription factor EB (TFEB) activation in vitro DE model [47]. The active form of vitamin D3, calcitriol, also augmented autophagy flux and inhibited apoptosis and inflammation via vitamin D receptor signaling pathway to prevent ocular injury and clinical complication caused by DE [48]. In addition, our previous research has shown that autophagy is involved in the pathogenesis of DED caused by subcutaneous injection of scopolamine in a controlled environment, the level of corneal autophagy increased but it did not continue to increase with the aggravation of the disease. Autophagy activators increased the level of corneal autophagy, and decreased the expression of inflammatory response mediators, thus can be used to treat DED [49]. It is gradually well-known that there are complex connections between autophagy and ocular surface inflammation.

### Corneal allograft rejection

Corneal transplantation is the most common performed type of tissue transplant globally, which can restore the visual function after severe corneal impairment [50]. Despite the cornea have the relative immune privilege, allogeneic rejection is the most common reason of corneal transplantation failure [51]. A relative study has proposed that collagen V was secreted by the corneal epithelium into the corneal stroma by EVs pathway, which meant that EV secretion is involved in the biomaterials-induced corneal regeneration [52]. Furthermore, MSC-Exos with co-delivery of siRNA against Fas receptor and miR-375 inhibitor successfully improved islet transplantation [53], making exosomes possible to promote immune tolerance of corneal grafts.

It has been described that kaempferol, a natural flavonoid, alleviated corneal allograft rejection by inducing autophagy to inhibit the activation of NOD-like receptor family, pyrin domain containing 3 (NLRP3) inflammasomes and macrophage polarization, which provided a novel therapeutic drug in immune rejection of the graft [54]. The enhanced autophagic could inhibit NLRP3 inflammasome activity and prolong the survival of murine corneal allografts [55].

### Corneal injury

The corneal wound healing process experiences the proliferation of corneal epithelium, stromal cells, and myofibroblast, the deposition of collagen, and the infiltration of inflammatory cells. It has been shown that exosomes played a role in corneal repair process and neovascularization by transporting C–C motif chemokine 2, C–X–C motif chemokine 5, thrombospondin-2, and latent-transforming growth factor beta-binding protein 1 [56]. Matrix Metalloproteinase 14 (MMP-14) participated in the formation of corneal neovascularization through the exosomal pathway and a series of related signal transduction pathways [57]. Corneal fibroblasts secreted exosomes could deliver MMP-14 to corneal endothelial cells and participated in the corneal neovascularization formation [58]. Exosomes derived from human corneal mesenchymal stem cells (cMSC-Exos) have significantly enhanced corneal wound closure, indicating that the process of ocular surface damage was promoted [59]. Those results indicated that the epithelial-secreted exosomes might serve as targets for potential therapeutic interventions in corneal injury and neovascularization. As a cell–cell delivery vehicle, exosomes can replace stem cell therapy and are currently a promising treatment method. Transplantation of umbilical cord mesenchymal stem cells (uMSCs) to Lumican mice (Lum −/−) participated in the communication between donor uMSC and recipient corneal cells, successfully restored corneal transparency and increase corneal tissue thickness [60]. In addition, relevant research has indicated that adipose-derived mesenchymal stem cells-derived exosomes were involved in regulating the cell viability of corneal stroma and the remodeling of extracellular matrix, which were expected to be applied to the treatment of corneal stromal damage [61]. EVs secreted from corneal stromal stem cells reduced visual scarring in murine corneal wounds by means of decreasing the levels of fibrotic genes collagen type III alpha 1 and actin alpha 2, preventing neutrophil infiltration, and restoring normal structure [62]. Remarkably, induced pluripotent stem cells-derived exosomes showed a better effect than MSC-Exos in treating corneal epithelial cells in vitro or corneal epithelial models in vivo [63].

In corneal injury mice caused by blue light stimulation, the ROS-NOD2-ATG16L1 signaling pathway might be participated in autophagy induction with the excessive expression of NOD2 on the ocular surface, which leads to corneal epithelial cell apoptosis and death [64]. By inhibiting the PI3K-Akt-mTOR pathway, S100 calcium binding protein A4 silencing had a positive effect on alkali-burned corneal injury. It suppressed the proliferation, migration, fibrosis, and invasion of rabbit corneal stromal cells, facilitated the autophagy and the differentiation of corneal cells [65]. In scratch injured HCECs, treatment with epidermal growth factor (EGF) could activate the transient receptor potential cation channel subfamily M member 2 pathway by upregulating reactive oxygen species (ROS)-induced calcium influx to activate mitochondrial autophagy and inhibit apoptosis, and finally promote the repairment of HCECs [66]. H_2_O_2_-induced corneal oxidative injury inhibited the autophagy process, and rapamycin, a common autophagy activator, could reverse and ameliorate the condition. That also revealed the relationship between of ROS and autophagy [67]. Cigarette smoke extract resulted in the accumulation and cytotoxicity of ubiquitinated proteins in HCECs caused by the autophagic impairment, likewise, this could be reversed by an autophagy activator, cysteamine [68].

### Keratitis

As one of infectious corneal diseases, fungal keratitis may result in blindness [69]. In Fusarium solani fungal keratitis (FK) mouse models, miR-223-3p might regulate autophagy via targeting ATG16L1 and was associated with the inflammatory response, which might be a potential therapeutic target [70]. Another study has demonstrated that the expression of miR-665-3p in the cornea was upregulated and autophagy flux was impaired when infected with Fusarium solani, thus miR-665-3p inhibitors might activate autophagic pathways and inhibit inflammation to cure FK [71]. The autophagy level in mice corneas increased after fumigatus infection, especially at 3 days. Contrary to the decreased levels of inflammatory cytokines IL-1β, interleukin-18 (IL-18), high mobility group box 1, TNF-α, and IL-10 after treating with rapamycin, increased levels of them after treating with 3-methyladenine or chloroquine, which meant that autophagy might become a novel target for relieving Aspergillus fumigatus FK [72]. Moreover, a more specific study has confirmed that the indispensable signaling pathway for the initiation of autophagy, cGAS-STING, was activated in HCECs infected with Aspergillus fumigatus [73]. Viral keratitis caused by human herpes simplex virus type-I as a result of neurotoxic factor reversed protein kinase R-mediated phosphorylation of eukaryotic initiation factor 2 alpha reduced the expression of the autophagy marker gene Beclin1, and inhibited the presentation of autophagy-mediated type II antigen to enhance the virulence [74, 75]. The expressions of apoptosis and inflammation, and the phosphorylation expression of ER stress-related proteins increased in exposed keratitis models of HCECs and C57BL/6 mice, indicate that autophagy might promote cell survival [76].

### Corneal dystrophy

Corneal dystrophy (CD) with the abnormal deposition of substances in the cornea, is sub-classified by the anatomic location affected: epithelial/subepithelial, epithelial-stromal, stromal, and endothelial dystrophies [77]. Corneal endothelial cells are attached to the descemet membrane, results in vision impairment and corneal edema when damage or loss [78, 79]. Previous study indicates that mesenchymal stem cell-derived extracellular vesicles down-regulate the endoplasmic reticulum stress-related genes, up-regulate the Akt pathway to inhibit the levels of apoptosis related caspase-3 activation in human corneal endothelial cells in vitro model, suggesting a potential therapeutic effect on corneal endothelial dystrophy [80].

Fuchs endothelial corneal dystrophy (FECD) is the most common corneal endothelial dystrophy, which is a genetically complex, heterogenous, age-related degenerative disease of corneal endothelial cells with a higher incidence in females. The activation of PINK1-Parkin-mediated mitophagy could degrade mitochondrial quality control proteins in FECD [81]. Lattice corneal dystrophy (LCD) is a degenerative disorder that causes loss of corneal transparency and eventually leads to loss of vision, and the reversion of the defective autophagic process in macrophages might be a therapeutic strategy for patients. The impairment of autophagic degradation of mutant transforming growth factor-β-induced protein (Mu TGFBIp) as a result of incomplete autophagy flux in macrophages, which prevented the further phagocytic activation and lead to LCD [82]. Thiel–Behnke corneal dystrophy (TBCD) caused by mutations of TGFB is an epithelial-stromal dystrophy and will be treated by the activation of autophagic flux and the amelioration of lysosomal function [83]. The pathological feature of granular corneal dystrophy type 2 (GCD2) is that mutant TGF-β deposits on the cornea with the defect of autophagy degradation in corneal fibroblasts, and TFEB has therapeutic potential in alleviating this situation to treat the disease [84]. Melatonin could enhance autophagy activation by relying on the mammalian target of rapamycin (mTOR) pathway and increasing the degradation of Mu TGFBIp. The effect was more obvious with the cotreatment of melatonin and rapamycin [85]. Lithium chloride (LiCl) increased autophagy in Mu TGFBI-overexpressing cells, inhibited the negative effects and partly recovered the cell viability in GCD [86]. Autophagy was triggered and autophagic flux was impaired in keratocytes of macular corneal dystrophy (Groenouw II type CD), which is a rare autosomal recessive-inherited condition characterized by progressive loss of vision, photophobia, and discomfort on the ocular surface [87].In the cornea of Slc4a11 knock out mouse model, mitochondrial ROS disrupted TFEB signaling causing impairment of autophagy and congenital hereditary endothelial dystrophy [88].

### Keratoconus

With corneal thinning and irregular astigmatism, keratoconus (KC) is a common eye disease and causes progressive loss of vision [89]. Impaired autophagy regulation or differential autophagic expression of its related proteins (LC3 and lysosome-associated membrane protein 1) under oxidative damage condition in the cornea might be involved in the pathogenesis and progression of KC [90]. The increased LC3 expression of corneal cells has been observed in KC, which also speculated that autophagy participated in KC [91].

## Research progress of exosomes and autophagy in retinal diseases

### Retinal vascular diseases

According to our previous research, in the retinal oxidative stress model, oxidative damage induced the autophagy of retinal astrocytes (RACs), up-regulated the pathways related to migration and proliferation and affected the function of endothelial cells ultimately. Exosomes derived from RACs were major factors during this process. Therefore, RACs released exosomes and the autophagic signals transmitted by them might adjust the survival, proliferation, and migration of retinal endothelial cells, thereby took part in the pathological mechanism of retinal vascular diseases [92]. Wnt inhibitory factor 1 could inhibit pathologic neovascularization, enable physiological revascularization of ischemic tissue, and mitigate retinal neuronal damage in oxygen-induced retinopathy mice by improving autophagic flux [93].

### Diabetic retinopathy

The pathological mechanism of DR is the increased levels of inflammatory cytokines in the retina, which often leads to vision loss in the elderly [94]. Relative research indicated that MSC-Exos equipped with miRNA-126 alleviated hyperglycemia-induced retinal inflammation by blocking the high mobility group box 1 signaling pathway [95].

DR is one of the serious complications of diabetes, as well as the predominant pathological manifestation of diabetic microangiopathy. The early characteristic change is that the loss of pericytes leads to retinal microvascular dysfunction. Autophagy could improve the viability of pericytes under certain stress conditions. In the state of hyperglycemia, the expression levels of LC3 and p62 proteins in retinal pigment epithelium cells (RPEs) changed, and the autophagic vesicles increased significantly to maintain the cellular environmental homeostasis [96]. Due to lysosomal membrane permeabilization, high glucose inhibited the autophagy and reduced its degradation. In the early stage of DR, knocked down the expression level of high mobility group box 1 would restore autophagy degradation, reduced the expression of vascular endothelial growth factor and inflammatory factors, finally avoided the apoptosis of RPEs [97]. In diabetes-induced mice with retinal degeneration, photoreceptor cell death occurred before the appearance of retinal vascular changes, which might be caused by the increased autophagic activity observed in these cells [98].

The crosstalk of exosomal secretion and autophagy have been revealed that affected on the treatment of DR. Thioredoxin regulated AMPK-mediated autophagy and exosome secretion to improve diabetes-induced photoreceptor cell degeneration, and might be a beneficial therapy to alleviate retinal photoreceptor cell degeneration and provide for new clinical therapeutic targets for DR [99].

### Retinal ischemia

Retinal ischemia causes retinal tissue damage and progressive degeneration of retina ganglion cells [100]. Intravitreal injection of MSC-Exos relieved the severity of retinal ischemia murine model without immunosuppression, which provided a novel non-cellular therapeutic style [101].

Angiogenic factor with G patch domain and forkhead-associated domain 1 has been shown to activate autophagy to promote angiogenesis for ischemic retinopathy and inhibition of PI3K/AKT/mTOR pathway [102]. In addition, rapamycin induced autophagy to decrease persistent gliosis and vascular endothelial growth factor (VEGF) synthesis, and modulated vascular and non-vascular alterations through direct effect on Müller glial cells, two relevant aspects of retinal ischemia [103].

### Age-related macular degeneration

AMD is a macular disease of the retina with unclear pathogenesis, which cause progressive damage to the patient’s vision [104]. One of the main pathogenic factors of AMD was suffering oxidative stress, the expression of vascular endothelial cell growth factor receptor and the release of exosomes were increased, especially exosome-associated marker protein CD63 and lysosome associated membrane glycoprotein 2 increased significantly in stressed RPEs [105], suggesting that exosomes are closely bound up with the occurrence of AMD. Oxidative stress might promote the angiogenesis ability of endothelial cells and an increased number of RPEs-derived exosomes, which may lead to neovascularization [106]. Studies have also indicated that with simulating the highly oxidative stress cell environment of neovascular AMD, up-regulation of exosomal proteins such as heat shock protein 70, cathepsin D, and cytokeratin 8 could be potential biomarkers and therapeutic targets for the diagnosis and treatment of AMD [107].

The damage to the autophagy-lysosomal degradation system of RPEs is one of the basic pathological features of AMD. When the ATP level in RPEs of AMD was decreased, the autophagic level was reduced [108]. At the same time, the accumulation of lipofuscin lead to the production of free radicals and cell apoptosis, further brought to RPEs dysfunction [109]. Inhibiting mTOR signal pathway of RPEs during oxidative stress could treat retinal degeneration in mouse age-related retinopathy models [110]. In the blue light-induced retinal degeneration model, cynaroside induced autophagy in vitro and in vivo to alleviate apoptosis and protect retinal tissues [111].

Exosomal secretion and autophagy have also been confirmed that influence the development of AMD. To be specific, autophagy induced by RPEs under the exosomal pressure would increase the autophagy levels in other cells, which might contribute to the formation of optic disk drusen (an early symptom of AMD) [105]. In addition, the hypoxic damage to cells altered autophagy and exosomal biogenesis. Treating RPEs with ATG7, a key protein that formed autophagosomes, would promote the release of exosomes containing VEGF receptor 2 and alleviate angiogenesis [112].

### Retinal detachment

The connection between autophagy and exosomes processes has been reflected in retinal detachment. It has been shown that MSC-Exos enhanced the autophagy level to inhibit the induction of inflammatory mediator factors TNF-α and IL-1β after neurosensory retina separation, which increased the survival rate of photoreceptor cells. Therefore, MSC-Exos developed a new therapeutic effect on retinal detachment [113].

## Conclusions

Visual impairment caused by ocular surface and retinal diseases is one of the essential public health issues which raise wide concern heat in society nowadays. When it comes to their most fundamental pathogenesis, the interaction between autophagy and exosomal biogenesis exerts influence on them. At the molecular level, autophagic proteins and complexes play crucial roles in exosomal biogenesis. At the organelle level, exosomal and autophagic pathways intersect at amphisomes and their contents have different consequences such as EVs release or lysosomal degradation. Growing evidence has shown that the cellular responses are closely related to exosomal biogenesis and autophagy to maintain cell homeostasis and to alleviate stress. In general, this review facilitates the perspective of the mechanisms of autophagy and exosomes in ocular surface and retinal diseases. It is already clear that the dynamic interaction between exosomal biogenesis and autophagy has important effects on normal physiology and disease condition. For this point, based on the complex and diverse mechanisms in this field of ophthalmology, the functions and mechanisms of exosomes and autophagy are mostly limited in fundus diseases including retinal vascular diseases, DR, and AMD, while diseases in other parts of the eye such as ocular surface and corneal diseases, cataracts, glaucoma require further investigation. Therefore, if we can better understand the complex mechanisms and adjustment processes, it can provide a new direction for the diagnosis and treatment of ophthalmological diseases.

## Data Availability

Figure 1 was created in part with Servier Medical Art (http://smart.servier.com), licensed under a Creative Common Attribution 3.0 Generic License (https://creativecommons.org/licenses/by/3.0/).

## Abbreviations

ALIX: Apoptosis-linked-gene-2 interacting protein X
AMD: Age-related macular degeneration
ATG5: Autophagy-related protein 5
CD: Corneal dystrophy
cMSC-Exos: Exosomes derived from corneal mesenchymal stem cells
DED: Dry eye disease
DR: Diabetic retinopathy
EGF: Epidermal growth factor
EMS: Endomembrane system
ER: Endoplasmic reticulum
ESCRT: Endosomal sorting complex required for transport
EVs: Extracellular vesicles
GCD2: Granular corneal dystrophy type 2
FK: Fungal keratitis
HCECs: Human corneal epithelial cells
ILVs: Intraluminal vesicles
IL-1 beta: Interleukin-1β
IL-6: Interleukin-6
IL-10: Interleukin-10
IL-18: Interleukin-18
KC: Keratoconus
LCD: Lattice corneal dystrophy
LC3B: Light chain protein 3B
MMP-14: Matrix metalloproteinase 14
MSC-Exos: Exosomes derived from mesenchymal stem cells
mTOR: Mammalian target of rapamycin
Mu TGFBIp: Mutant transforming growth factor-β-induced protein
MVB: Multivesicular bodies
NLRP3: NOD-like receptor family, pyrin domain containing 3
OSDs: Ocular surface diseases
PIK3C3: Mammalian class III PI3K
RAC: Retinal astrocytes
SS: Sjogren’s syndrome
RPEs: Retinal pigment epithelial cells
SS-DE: Sjögren syndrome dry eye
TBCD: Thiel–Behnke corneal dystrophy
TFEB: Transcription factor EB
TNF-α: Tumor necrosis factor-alpha
TGF-β: Transforming growth factor-beta
uMSC: Umbilical cord mesenchymal stem cell
VEGF: Vascular endothelial growth factor
